# Facile Fabrication of Spherical Nanoparticle-Tipped AFM Probes for Plasmonic Applications

**DOI:** 10.1002/ppsc.201400104

**Published:** 2014-08-12

**Authors:** Alan Sanders, Liwu Zhang, Richard W Bowman, Lars O Herrmann, Jeremy J Baumberg

**Affiliations:** NanoPhotonics Centre, Department of Physics, University of CambridgeCambridge, CB3 0HE, UK E-mail: jjb12@cam.ac.uk

**Keywords:** electrodeposition, plasmonics, tips, nanoparticles, TERS

Recently, plasmonics has attracted extensive attention for its ability to enhance evanescent electric fields at nanometer spatial scales. Much research has become dedicated to the use of sharp metal tips as nanoscale optical antennae, exploiting surface plasmon resonances. Unlike planar metallic surfaces, tips support localized surface plasmons (LSPs) and benefit from strong field localization at the tip apex. This gives rise to a high spatial resolution when applied to existing plasmonic applications such as surface-enhanced Raman spectroscopy (SERS), leading to tip-enhanced Raman spectroscopy (TERS), which allows for high-resolution chemical mapping of surfaces.

However, due to their extended nature, real tip structures behave very differently to isolated plasmonic nanostructures such as metallic nanoparticles and nanorods. Though the plasmon resonances of tips can be tuned via the aspect ratio[1–3] and the refractive index of the medium inside the tip,[4,5] the behavior of such plasmonic modes remains very different to isolated nanostructures. Nanostructuring a tip has been seen to improve its effectiveness in plasmonic systems,[6,7] thus tips are now often modified prior to use.[8,9]

Although tips come in many forms, the standardized tips of AFM probes are especially useful due to their variety of applications throughout scanning microscopy. It is thus desirable to produce AFM tips capable of effectively supporting plasmon resonances, in order to take advantage of the high resolution they enable in scanning microscopy.[10–14] Nanostructured AFM tips with vacuum-processed individual spherical tips (NanoTools GMBH) have recently been used to measure quantum effects in plasmonic systems.[9] Further evidence for the effectiveness of spherical nanoparticle tips for plasmonics has been reported, with strongly enhanced TERS spectra achieved due to resonant excitation of LSPs in the tip nanostructures.[6–8] We therefore focus here on the spherical tip geometry.

Fabrication of spherical tips for plasmonics can be achieved by mounting single nanoparticles onto the apex of a tip. This concept has been reported numerous times over the last decade,[15] beginning with the use of fibers as mounting structures[16–19] and progressing onto the use of scanning probe microscopy (SPM) tips.[6,8,13,20,21] While mounting nanoparticles onto SPM tips is more difficult than with fibers the additional capabilities of the SPM tip have made such tips desirable. However, these tips typically require complicated assembly processes to precisely secure a single nanoparticle at the apex of the tip, greatly increasing their fabrication time and costs. More recent methods have attempted to address the complexity issue by directly depositing nanoparticles onto the apex by exploiting localized chemical reactions. However, these techniques have still been limited by cost or required specialist equipment,[18,20] incompatibility with SPM probes,[7,17] or constraints on nanoparticle growth either in size[22] or material.[6] It remains a challenge to find a simple and efficient method for reliably producing spherical nanoparticle tips. Here, we provide a simple and efficient method for reliably producing spherical nanoparticle tips using apex-selected electrochemical growth.

Electrochemical deposition is highly suited to the tip geometry because of the large field enhancement localized at the sharp point. Due to the significantly reduced radius of curvature at the tip apex, the equipotential surface resulting from an applied voltage leads to compression of field lines in the region. This strongly increases the field amplitude at the tip apex and is known as the lightning rod effect. Under such conditions, the rate of electrochemical reactions is significantly increased around the vicinity of the tip apex. By exploiting this localized field enhancement, it is possible to grow a spherical nanoparticle directly onto the tip apex. While use of the lightning rod effect for electrochemical growth has been used to grow dense forests of nanoparticles[23,24] it has yet to be applied to the fabrication of single nanoparticles at the tips apex. A disadvantage of electrochemical deposition is that it is difficult to selectively grow nanoparticles on the tips. Here by using single-pulse high-field electrochemical growth, we selectively grow nanoparticles on the tip apex. Using this approach, we demonstrate an efficient, high-throughput technique for reliably producing metallic spherical nanoparticle tips using only a simple electrochemical cell.

Fabrication of Au spherical nanoparticle (AuNP) tips onto commercial AFM probes is achieved using single-pulse high-field electrochemical growth. Conductive coatings are required for the electrochemical reaction, therefore Au- and Pt-coated AFM tips are used. Tips are pretreated with oxygen plasma to remove organic contaminants from the surface prior to growth. A simplified two-electrode system is employed for growth since both the cell geometry and electrodeposition solution are kept the same between fabrications. AFM probes are attached to fluorine-doped tin oxide (FTO) conductive glass, used as a working electrode, opposite a Pt wire counter-electrode (**Figure** **1**a,b). Simultaneous fabrication of both Au and Pt tips is carried out by contacting multiple tips, closely spaced side-by-side on the same FTO surface. Linear sweep voltammetry of a single AFM tip in ECF60 (Figure 1c) shows that Au growth starts at −1.1 V.

**Figure 1:**
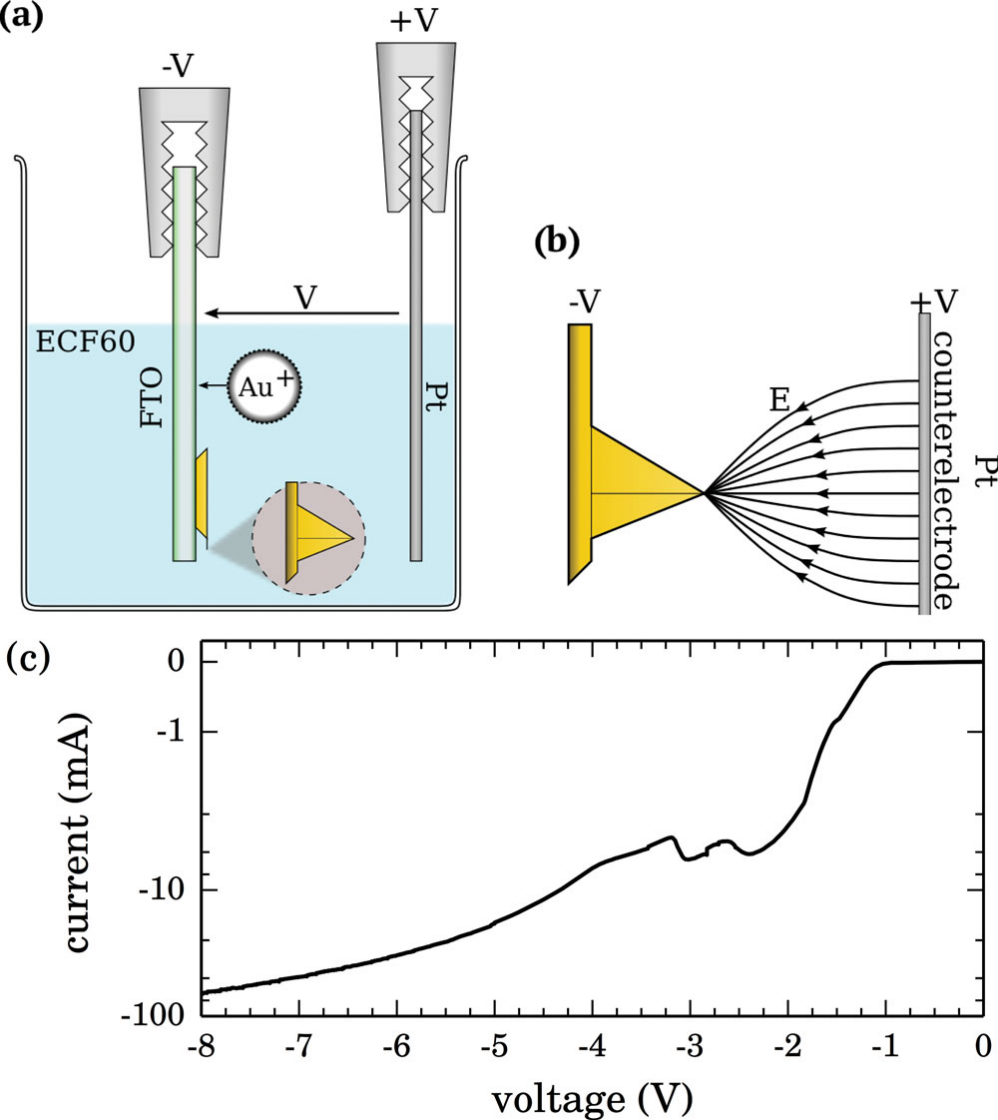
a) Electrochemical cell for growth of Au onto the apex of an AFM tip. b) Termination of field lines at tip apex due to the lightning rod effect enhances localized electrochemical growth for single NP growth. c) Linear sweep voltammetry of a single AFM cantilever held by an Al clamp cathode out of solution (replacing the standard FTO electrode to show growth characteristics of only the tip).

A single high-voltage pulse is applied to nucleate and grow a single AuNP at the tip apex. Due to the large field amplitude at the tip apex, field lines from across the cell terminate at the apex inducing ions to drift towards the tip (Figure 1b). Multiple combinations of applied voltage and pulse time were investigated to optimize growth parameters. The growth of Au onto the AFM tip is confirmed by current dynamics, revealing a 2–3 ms initiation followed by relaxation to continuous diffusion-limited growth within a few tens of milliseconds.

Scanning electron microscopy (SEM) images of four tip samples fabricated at −8 V are shown in **Figure** **2**, three of which were fabricated simultaneously on one FTO glass slide, to show the effects of tip pretreatment and exposure time in the applied field. These images demonstrate that spherical AuNP tips can be reliably fabricated using the proposed electrodeposition procedure. Spherical AuNP growth diameters between 150 and 450 nm are achieved using 100–200 ms pulses across the electrochemical cell on both Pt and Au tips. Evidence of growth localization to the high field regions is clearly exhibited by the formation of spherical AuNPs at the tip apex. The morphologies obtained differ with and without plasma pretreatment. Using tips as supplied leads to a smooth sphere at the tip apex resulting from the lightning rod effect, followed by a broad neck and semi-uniform Au coating across the exposed surfaces of the tip (Figure 2a). Plasma treatment removes organic contamination and can also oxidize the surface.[25,26] The formation of an insulating metal oxide layer prevents growth on surfaces, limiting growth only to sharp regions with a small radius of curvature (Figure 2b–d). These regions remain conductive and the electric field is large and highly localized. Because Au is more difficult to oxidize than Pt, the shielding effect is different for plasma-treated Au-coated AFM probes, which exhibit significant localized nanoparticle growth on all exposed surfaces (Figure 2c). A longer pulse time can result in a larger diameter of spherical AuNP, as shown in Figure 2b,d. A 200 ms pulse growth leads to a spherical AuNP on the Pt tip with a diameter of ≈450 nm (Figure 2b), while the diameter of the AuNP is 150 nm with a 100 ms pulse (Figure 2d).

**Figure 2:**
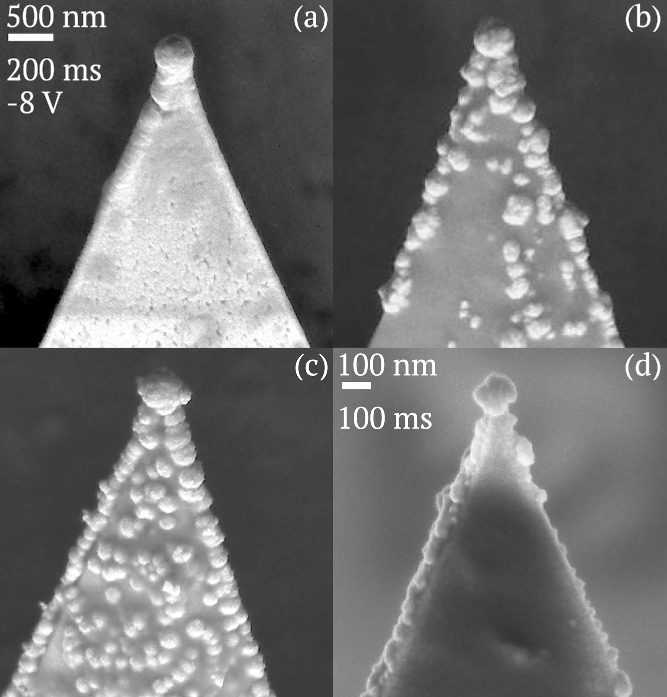
Comparison of AuNP-tipped AFM probes, fabricated on various base structures using −8 V pulses of different lengths. The first three tips were produced simultaneously using a 200 ms pulse on a) a commercial Pt tip with no pretreatment, b) a plasma-treated Pt tip, c) a plasma-treated Au tip. d) Duplicate spherical tip produced separately on a plasma-treated Pt tip using a 100 ms pulse.

To investigate the dependence of fabricated tip morphology on the pulsed voltage across the electrochemical cell, the growth of AuNP on Pt tips at different applied voltages is further studied. SEM images of such fabrications along with corresponding current transients are shown in **Figure** **3**. Images show that apex-selected growth occurs only once the voltage is more negative than −3 V. This voltage dependence is attributed to changes in deposition mechanism with field strength.

**Figure 3:**
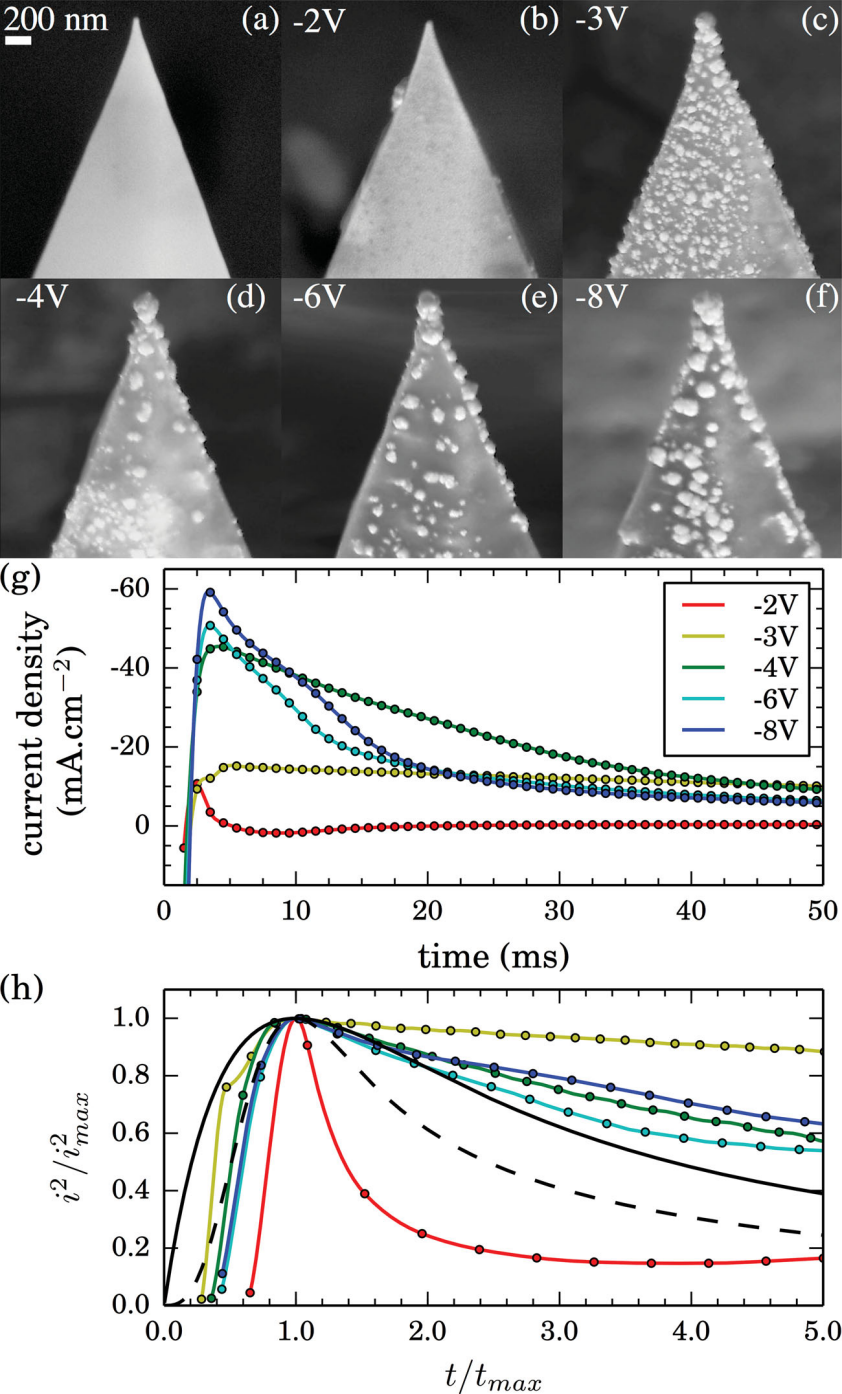
Comparison between a) a standard Pt AFM tip and b–f) AuNP growth on plasma-treated Pt tips using 150 ms pulses for voltages between −8 and −3 V and a 500 ms pulse at −2 V. This shows the change in deposition mechanism as the magnitude of applied field strength is increased, with no spherical growths above −3 V irrespective of exposure time. g) Current transients from current traces measured during fabrication of tips shown in (b-f), offset by the saturation current density (−10, −31, −34, −78, −142 mA cm^−2^, respectively). h) Variable-independent reduced current transients (colored lines) measured at various applied voltages during fabrication compared with theoretical curves for progressive (dashed) and instantaneous (solid) nucleation current transients.[27]

At low voltages (≥–2 V), electrodeposition forms smooth film coatings (Figure 3b) similar to direct-current electrodeposition. Under these conditions, growth is dominant over nucleation and the field profile caused by the lightning rod effect is eventually evened out, yielding smooth rounded tips. Even with 500 ms exposure time, no apex-selective growth is observed despite the charge transfer being equivalent to AuNP tip growths at more negative potentials.

Increased nucleation is observed at −3 V where spherical tip growth is initiated under a progressive nucleation mechanism with some preferential growth at the tip apex, as evidenced by the large number of nucleated particles and variation in particle size (Figure 3c). For more negative voltages (≤–4 V) nucleation becomes more selective, leading to clean surfaces and improved apex-selected growth (Figure 3d–f). This is due to a transition to instantaneous nucleation, in which a fixed number of particles nucleate at selected active sites on application of a field.[27] This occurs preferentially at sharp edges where the field is highest. Further selection may occur through depletion of ions in the vicinity of the growing tip, preventing additional growth sites. This helps to produce an isolated AuNP at the tip apex. Increasing the magnitude of the voltage increases the number of active sites available for nucleation and more of the tip surface surpasses the field threshold for instantaneous nucleation. Hence, using less negative voltages within the instantaneous nucleation regime reduces the number of active nucleation sites leading to cleaner spherical growth at the tip apex.

This changeover in nucleation mechanisms is also observed in current transients (Figure 3g) as the shape distinctly changes when decreasing the voltage below −2 V. The largest change in transient shape occurs at −3 V, indicating the onset of short-time-scale nucleation. Elongation of the transient time is likely caused by contribution to the current from progressive particle nucleation throughout the exposure. The large short-time-scale transients observed for potentials more negative than −4 V support the hypothesis of an instantaneous nucleation mechanism as the fast current decay indicates the saturation of all active sites leaving only diffusion-limited growth.

The influence of instantaneous nucleation for isolated AuNP tip growth is further evident in comparisons to theoretical reduced current transients for diffusion-limited progressive and instantaneous nucleation (Figure 3h) developed by Scharifker and Hills (SH model).[28] Reduced current transients are normalized to (*i*/*i*_max_)^2^ and plotted against *t*/*t*_max_ to remove variable dependencies, where *i*_max_ and *t*_max_ represent the peak current and corresponding time. In general, for less negative deposition potentials (−2 V), nucleation resembles more closely progressive nucleation and growth, while at more negative potentials (<−4 V) it resembles more closely instantaneous nucleation. This correlates well with the SEM images shown in Figure 3d–f. Variations from theory occur due to the variable field profile present across the tip leading to localized instances of both progressive and instantaneous nucleation contributing to the overall current. The SH model is built upon assumptions such as random nucleation and provides valid results only for the limiting cases of only progressive or instantaneous nucleation for which the validity fails in such a selectively grown system.[29]

Electrochemically grown AuNP tips are characterized in a custom-built optical microscope with two opposing AFM probe mounts (described previously).[9] Dark-field and Raman spectroscopies are used to demonstrate the plasmonic properties of fabricated tips. Fabricated AuNP AFM tips are mounted opposite a benzenethiol-coated sharp Au AFM tip in a tip-to-tip configuration, mimicking a plasmonic bow-tie antenna (**Figure** **4**a). This configuration is used to obtain good optical access to the dimer gap for spectroscopically probing its plasmonic properties. The gap between tips is reduced to 1 nm, as set by the thickness of the assembled molecular layer, and illuminated with 638 nm light. Benzenethiol (BTh) is used as a Raman marker for measuring the relative field enhancement of AuNP tips due to its strong Raman response and well-known spectra.[30,31]

**Figure 4:**
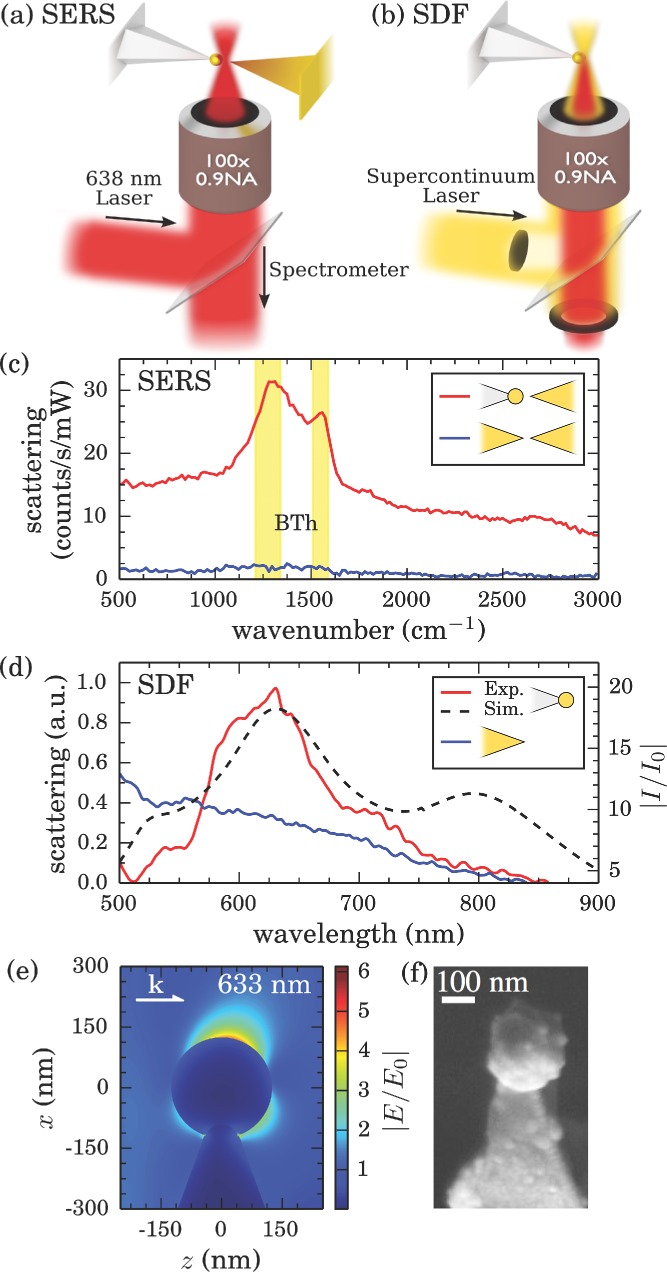
Experimental geometry for a) SERS measurements and b) dark-field spectroscopy. A 125 nm radius spherical AuNP grown onto a Pt-coated AFM tip is spectroscopically studied using a supercontinuum laser in a dark-field configuration. The tip is then brought within 1 nm of a benzenethiol-coated sharp Au tip under 638 nm illumination to measure SERS spectra. c) Tip-enhanced Raman spectra of a benzenethiol-coated Au AFM probe brought close to the AuNP tip (red) compared with a sharp Au AFM tip (blue). d) Dark-field optical scattering of AuNP (red) and sharp Au (blue) AFM tips, with calculated relative intensity enhancement 0.5 nm from the AuNP tip apex (dashed). e) Calculated field enhancement profile for a 125 nm radius AuNP at the end of a 1500 nm long Pt tip (50 nm neck joint width) under longitudinally polarized plane wave illumination at 633 nm. f) SEM of the 125 nm AuNP tip.

A 125 nm radius spherical AuNP tip grown at −8 V for 150 ms is used to demonstrate the augmented plasmonic properties when using modified tips. Raman spectra of BTh molecules in the tip dimer gap are greatly enhanced when using a AuNP tip in place of a sharp Au tip (Figure 4c). As the same spectrometer is used for both broadband scattering spectra (300–1100 nm) and SERS spectra, its restricted spectral resolution blurs the characteristic multiple Raman peaks of BTh between 1000 and 1600 cm^−1^. However, the resulting observation of two broad peaks in this region affirms the presence of BTh in the gap between tips. The background signal is also enhanced across a broad bandwidth, as is typical for SERS.[30]

Supercontinuum dark-field scattering spectra (Figure 4b,d) taken of a single, isolated AuNP tip prior to SERS measurements show that the increased Raman enhancement is due to excitation of a LSP around *λ* = 630 nm, not present in sharp Au tips. The observed resonance between 600 and 700 nm is characteristic of these AuNP tips and is observed in multiple fabricated tips and commercial AuNP tips. The experimental spectrum is in good agreement with boundary element calculations of the near-field enhancement at the AuNP tip apex with a visible plasmon resonance observed across the AuNP (Figure 4d,e). Coupling between this LSP in the AuNP tip with a BTh-coated sharp Au tip forms a confined gap plasmon mode. Since coupling is between higher order modes in the sharp Au tip, shifting of this resonance as a function of gap size is weak.[32] Illuminating on resonance with the gap plasmon therefore greatly increases the Raman response by a factor of order 30. This corresponds to a relative SERS enhancement of 12 when using a AuNP tip compared with a sharp Au tip after taking into account the confinement and mode volume of an LSP to the gap in each case.[32] Since the LSP is laterally confined to only 7 nm within this gap, the enhanced Raman signal is the result of scattering contributions from only a very small number of molecules. This leads to lower bound estimates for the absolute SERS enhancements of 1.9 × 10^5^ for the AuNP tip and 1.6 × 10^4^ for a sharp Au tip. Though absolute estimates are not as high as expected, the relative SERS enhancement observed with the AuNP tip is indeed comparable to previously reported results.[6]

These optical measurements confirm that AuNP tips provide increased field enhancement compared to sharp Au tips due to a strong LSP excitation. Lack of any strong peaks around 600 nm in dark-field spectra of sharp Au tips suggests that any plasmons present are weakly coupled and do not scatter strongly in this illumination geometry. Such plasmons may still couple with the opposing tip to form a gap mode but reduced localization results in a lower field enhancement. On the other hand, our AuNP tips are well suited to high enhancements when illuminated at the appropriate plasmonic resonances.

While a number of plasmonic probes have been developed recently, several useful features are obtained here. By using standard AFM probes as a basis, these AuNP tips maintain their functionality as AFM probes for force microscopy, as demonstrated by capacitively-driven tapping mode AFM scanning (**Figure** **5**), which is used to align the tips. Here, one tip is driven by a periodic voltage, which causes it to oscillate due to the time-varying capacitance between tips. The driven tip is scanned at long range and constant height over the opposing tip while measuring the separation-dependent cantilever oscillation. The metallic coating of these tips also allows for simultaneous electrical measurements while performing optical and AFM force measurements. These tips therefore function as standard electrical AFM probes with added plasmonic functionality.

**Figure 5:**
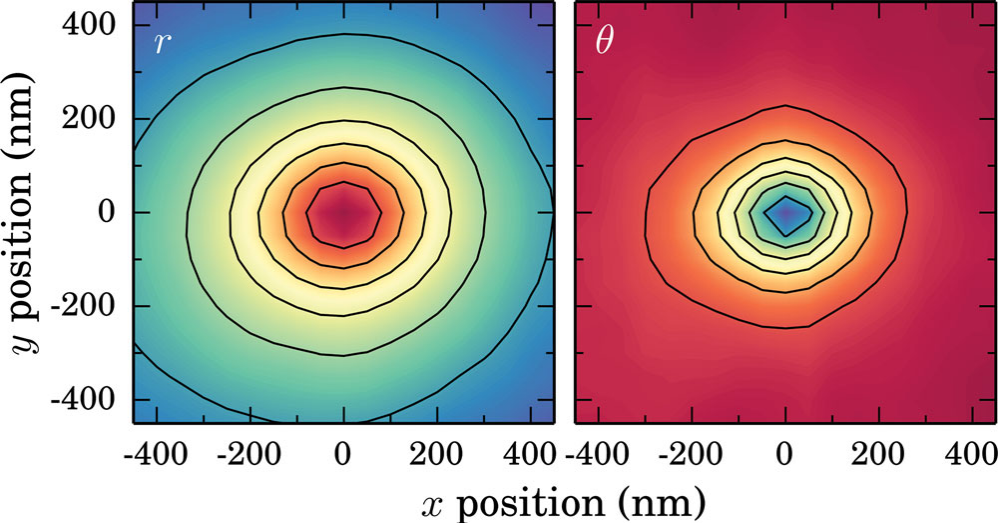
AFM tapping-mode scans of one tip by the other, using capacitance mode tracked in both (left) amplitude and (right) phase of cantilever vibrations, and showing 200 nm measured spatial width of AuNP probe.

The repeatability of such fabrication is excellent, with plasmon resonances in the same spectral location. Furthermore, such tips also show excellent resistance to damage at the tip apex after multiple surface contacts, though surfaces do become deformed after heavy use. Their robust nature is attributed to the direct growth of the AuNP root across the pyramidal tip end. This is a significant improvement over AFM tips with nanoparticles attached in which the spheres break from the tip and adhere to the contact surface after only a few contact cycles, and are not robust. This may also make them suitable for nano-indentation studies.

Several advantages emerge from this apex-selective single nanoparticle electrochemical growth. Simultaneous nanoparticle growth on many tips ensures a high-throughput process, while morphology and size are controlled by voltage and time. This provides a viable method for producing tips capable of expanding the user-base of plasmonics and furthering research into applications of TERS and SNOM (scanning near-field optical microscopy). Further applications are envisaged, for instance in plasmonic optical trapping[33] because the tips in the present geometry can conveniently act as a heat sink reducing the problematic optical heating observed, and resulting thermal damage. Furthermore, the spherical nanoparticle growth method introduced here is not restricted to specific metals, and many different composite systems can be created with this apex-localized growth technique. For example, silver nanoparticle tips give a larger optical response and field enhancement under visible illumination (although oxidation is sometimes an issue).

In conclusion, we have demonstrated a simple, fast method for the reliable growth of plasmonic spherical AuNP AFM tips. We showed this capability through measurements of dark-field scattering and SERS on controlled tip dimers at nanoscale separations.

## Experimental Section

*Fabrication and Characterization*: AFM Tips used are BudgetSensors ContGB and ContE models. FTO glasses, used as conductive substrates, are cleaned through sonication in 10 min steps using deionized water, ethanol, and finally acetone. AFM tips are pretreated for 20 min with oxygen plasma to remove organic contaminants from the surface prior to growth. A two-electrode configuration is used for the growth on an AutoLab potentiostat (PGSTAT 302N). AFM probes are attached onto an FTO conductive glass as a working electrode, while a Pt wire is employed as the counter-electrode, spaced 10 mm apart. Metalor ECF60 is used as the electroplating solution with no additives. The SEM measurements are carried out on a LEO GEMINI 1530VP FEG-SEM Scanning Electron Microscope. Current measurements are interpolated to create the smooth line observed behind data points in Figure 3g. Current transients (Figure 3h) are analyzed by first making a quadratic interpolation of experimental data points and then applying the *i*^2^/*i*_max_^2^ vs *t*/*t*_max_ transformation to extract the reduced current transients. This approach is used due to the limited number of points available in the peak region.

*TERS Measurements*: Benzenethiol (VWR International Thiophenol for synthesis) is diluted to 5 × 10^−3^ m solutions into ethanol (Sigma-Aldrich). Tips for use as SERS substrates are prepared by coating a monolayer of BTh onto the surface. This is achieved by submerging a standard Au-coated AFM tip in 100 × 10^−3^ m ethanolic BTh solution for 1 min followed by rinsing with ethanol and drying in nitrogen. This is repeated five times to ensure complete monolayer coverage. Tips for use as plasmonic probes are not coated in BTh. Tips are mounted onto three-axis nanopositioner stages in a custom-built optical microscope with dual-opposing AFM probe mounts and brought into axial alignment using a capacitively-driven tapping mode AFM alignment technique. The alignment scan is performed with a 10 V applied bias at 6.5 kHz to resonantly oscillate the contact-mode AFM cantilever at 13 kHz.[34] Repeated long-range scans are performed as the gap size is reduced from ≈1 μm to 100 nm until the signal-to-noise is large enough to confirm alignment without causing any potential tapping damage to tips. Once aligned, the gap size is reduced to around 1 nm, limited by the thickness of the assembled BTh molecular layer, and illuminated through a 100× 0.9 NA objective with 3 mW (1.9 MW cm^−2^) of 638 nm laser light incident on the gap, polarized along the tip axis. Scattered light is collected through the same objective and confocally localized. Raman spectra are filtered using a 650 nm long-pass filter prior to dispersion in a spectrometer. Contact dynamics confirming that tips come into contact while separated by a BTh layer are measured using in-built AFM laser deflection from the cantilever of the approaching tip. Near-field distributions were calculated using the full electrodynamic boundary-element method.[35,36] The ball tip was modeled as a Pt cone with half-angle 20° with a 250 nm diameter AuNP attached to its end. The neck radius between ball and tip was 50 nm. The tip was illuminated with a plane wave polarized along the tip axis.

*Field Enhancement Calculations*: Field enhancements are estimated by first calculating the mode volumes of plasmonic gap modes. The lateral width of a gap plasmon mode[32] is given by


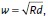
(1)

where *R* is the effective radius of the particles, 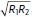, in the plasmonic dimer, and *d* is the width of the gap separating particles. This results in lateral mode widths of 4.5 nm for the sharp Au tip of 20 nm radius and 7.1 nm for a 125 nm AuNP tip. Assuming a cylindrical gap mode yields mode volumes of 15.7 and 39.3 nm^3^, respectively. These define the near-field contribution to Raman scattering and a relative field enhancement is obtained using


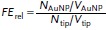
(2)

where *N* is the Raman signal counts and *V* is the mode volume. This evaluates to 12. Lower limit absolute field enhancements are estimated using


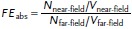
(3)

where *N*_far–field_ is assumed to be 0.1 counts/s/mW from the noise levels since signals are below the signal to noise level and *V*_far–field_ is assumed to be 25 000 nm^3^ based upon the surface of a conical tip exposed to the focal volume of a diffraction limited spot (*d* = 412 nm at *λ* = 638 nm). This expression yields absolute field enhancements of 1.9 × 10^5^ for an 80 nm AuNP tip and 1.6 × 10^4^ for a sharp Au tip.

## Supporting Information

As a service to our authors and readers, this journal provides supporting information supplied by the authors. Such materials are peer reviewed and may be re-organized for online delivery, but are not copy-edited or typeset. Technical support issues arising from supporting information (other than missing files) should be addressed to the authors.
