# Supplementary material for: Facile Fabrication of Spherical Nanoparticle-Tipped AFM Probes for Plasmonic Applications
Source: Part Part Syst Charact. 2014 Aug 12;32(2):182–7. doi: 10.1002/ppsc.201400104 (PMC4511392; doi:10.1002/ppsc.201400104)
Supplement: Supplementary file 1 — Supplementary [file ppsc0032-0182-sd1.pdf]

Copyright WILEY-VCH Verlag GmbH & Co. KGaA, 69469 Weinheim, Germany, 2014.

Particle
& Particle Systems Characterization

Supporting Information

for *Part. Part. Syst. Charact.,* DOI: 10.1002/ppsc.201400104

Facile Fabrication of Spherical Nanoparticle-Tipped AFM Probes for Plasmonic Applications

*Alan Sanders, Liwu Zhang, Richard W. Bowman, Lars O. Herrmann, and Jeremy J. Baumberg**

Supporting Information

Facile fabrication of spherical nanoparticle-tipped AFM probes for plasmonic applications

*Alan Sanders, Liwu Zhang, Richard W. Bowman, Lars O. Herrmann and Jeremy J. Baumberg**

A. Sanders, Dr. L. Zhang, Dr. R.W. Bowman, L.O. Herrmann, Prof. J.J. Baumberg
NanoPhotonics Centre, Department of Physics, University of Cambridge, Cambridge, CB3 0HE, UK
E-mail: jjb12@cam.ac.uk

Apex-selective growth of AuNP tips requires large, negative voltages to initiate and saturate nucleation, otherwise smooth growth occurs. This evens out the lightning rod profile. An example of this is shown in Figure S1.

Reduced current transients are compared to two extremities of growth, as given by the SH model of nucleation. The SH model for dimensionless current transients during progressive and instantaneous nucleation (plotted in Figure 3h) are given by,

$$\left(\frac{i}{i_{max}}\right)^2_{inst} = 1.9542\left(\frac{t_{max}}{t}\right)\left\{1 - \exp\left[-1.2564\left(\frac{t}{t_{max}}\right)\right]\right\}^2,$$

$$\left(\frac{i}{i_{max}}\right)^2_{prog} = 1.2254\left(\frac{t_{max}}{t}\right)\left\{1 - \exp\left[-2.3367\left(\frac{t}{t_{max}}\right)^2\right]\right\}^2.$$

where i is the current, t is the time, and i_{max} and t_{max} are the peak current and corresponding time, respectively. These functions are widely used in electrodeposition to determine nucleation characteristics and are displayed on Figure 3b to compare to experimentally measured dimensionless current transients.

The robustness of AuNP tips is evident from the SEM images before and after use in the custom-built AFM microscope (Figure S2).

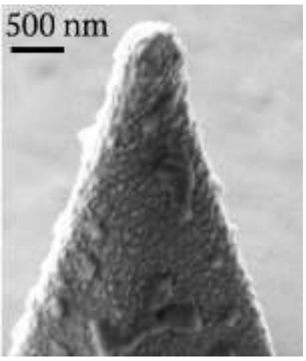

Figure S1. A tip fabricated at −2 V for 2.4 s showing the evening out of the lightning rod profile at long times and less negative voltages.

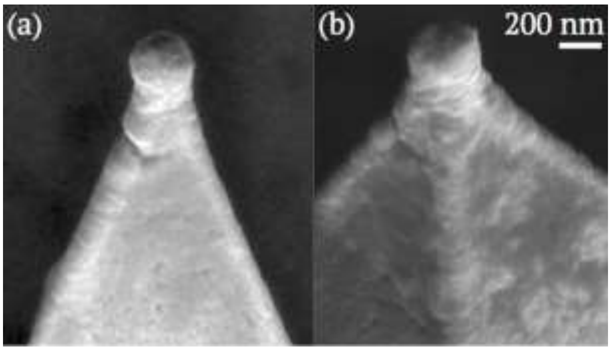

Figure S2. Before and after images of the same AuNP tip after heavy use showing the robustness of electrochemically grown tips.
